# Evaluation of pre- and post-surgery reading ability in patients with epiretinal membrane: a prospective observational study

**DOI:** 10.1186/s12886-020-01364-6

**Published:** 2020-03-10

**Authors:** Hiroki Mieno, Kentaro Kojima, Kazuhito Yoneda, Fumie Kinoshita, Rentaro Mizuno, Shinnosuke Nakaji, Chie Sotozono

**Affiliations:** 1 Department of Ophthalmology, Kyoto Prefectural University of Medicine, 465 Kajii-cho, Hirokoji-agaru, Kawaramachi-dori, Kamigyo-ku, Kyoto, 602-0841 Japan; 2 Department of Biostatistics, Graduate School of Medical Science, Kyoto Prefectural University of Medicine, Kyoto, Japan; 3 Department of Advanced Medicine, Nagoya University Hospital, Nagoya, Japan

**Keywords:** Epiretinal membrane (ERM), Vitrectomy, Reading ability, MinnesotaReading (MNREAD), Metamorphopsia, Metamorphopsia charts (M-CHARTS)

## Abstract

**Background:**

This study aimed to investigate the pre- and post-surgery reading ability in patients with idiopathic epiretinal membrane (ERM) to evaluate whether measurement of reading performance is a helpful test in addition to visual acuity (VA) as an assessment measure.

**Methods:**

This prospective observational study involved 42 eyes of 40 patients with idiopathic ERM. Best-corrected visual acuity (BCVA), reading ability, and metamorphopsia score were evaluated at baseline and at 3, 6, and 12 months post-surgery. As the outcome measure, the reading ability of each patient (i.e., overall performance) was examined with MNREAD-J, the Japanese version of the MNREAD reading acuity (RA) charts, to determine RA, critical print size (CPS), and maximum reading speed (MRS). Generally, a difference of 0.2 logMAR or more is considered a significant change in BCVA. Thus, as a subgroup analysis, we additionally evaluated the BCVA and reading ability of the patients with a BCVA difference of 0.1 logMAR or less between at baseline and at 12 months post-surgery.

**Results:**

Relative to their values at baseline, the subjects exhibited significantly improved BCVA, RA, and CPS throughout the post-surgery examination period (*P* < 0.001) and significantly improved MRS at 12 months post-surgery (*P =* 0.04). No significant change in the vertical metamorphopsia score was observed throughout the post-surgery follow-up period. However, and compared to the value at baseline, significant improvements in the horizontal metamorphopsia score were observed at 3, 6 (*P* < 0.05), and 12 months (*P* < 0.001) post-surgery. In the subgroup analysis of the 23 eyes that exhibited a BCVA improvement of 0.1 logMAR or less, the median BCVA did not change, but the median RA and CPS improved by 0.2 logMAR.

**Conclusions:**

Our findings showed that the surgical removal of ERM improves reading ability, even when the BCVA score does not improve. The measurement of reading performance appears to be a helpful test in addition to VA as a measure for assessing the surgical removal of ERM.

## Background

Epiretinal membrane (ERM) is an eye disease that is typically characterized by the proliferation of abnormal tissues on the surface of the central retina [1]. Once the ERM begins to contract on the macula and distort retinal cell layers, it often results in symptoms of visual disturbance or metamorphopsia, a type of defect that reportedly impairs the quality of vision, irrespective of visual acuity (VA) [2]. Thus, in ERM patients, VA examination alone may be insufficient for assessing the level of inconvenience that the disease imposes on their daily activities.

Because reading is one of the most important daily activities that relies on visual function, reading ability greatly influences a person’s quality of life [3–5]. Unlike VA, reading ability is a psychophysical measure consisting of two components, namely, reading speed (characters per minute) and reading acuity (RA), which refers to the smallest print size that a patient can resolve. To evaluate reading ability, the Minnesota Reading (MNREAD) charts are widely used [6–12] because they allow for simultaneous acquisition of the abovementioned objective reading parameters. Minnesota Reading charts have reportedly been used to assess the reading ability of both low-vision patients [13] and patients with a relatively high VA, as well as the reading ability of patients post cataract surgery [7, 10]. For countries in which English is not the native language, local-language-specific versions of MNREAD are now available that are comparable to the original English-language version, and numerous studies have reported the evaluation of reading ability with the use of these versions [6–9, 12].

Since the development of optical coherence tomography (OCT), a technique that enables practitioners to perform detailed observations of the macula, the diagnosis of ERM has considerably improved. The standard treatment for symptomatic ERM is vitrectomy combined with ERM removal. The surgical results have been favorable, especially since the development of less-invasive micro-incision vitreous surgery (MIVS). Since the trans-conjunctival 25-gauge (G) procedure was first reported in 2002 [14, 15], MIVS has rapidly become widely used, and recently, 27G systems have become available commercially [16]. Nonvitrectomizing vitreous surgery (NVS) has been also reported as an alternative surgical procedure to remove ERM without removing the vitreous [17]. Given that it is now possible to make an accurate preoperative diagnosis and perform less-invasive surgery, the surgical indication of ERM has expanded in recent years [18]. However, the trend of earlier indication for surgery has raised the question of whether performing surgery in ERM patients with relatively good preoperative VA is actually beneficial, thus indicating the need for other visual parameters, such as reading ability.

The present study aimed to investigate reading ability with MNREAD charts pre and post vitreous surgery to evaluate whether measurement of reading performance is helpful in addition to VA as an assessment measure.

## Methods

This prospective observational study was conducted in accordance with the tenets set forth in the Declaration of Helsinki, and prior written informed consent was obtained from each participant. The study protocols were approved by the Institutional Review Board of Kyoto Prefectural University of Medicine, Kyoto, Japan (Approval No.: RBMR-C-1217).

### Patients

The study involved 42 eyes of 40 patients who had undergone pars plana vitrectomy and membrane peeling for an idiopathic ERM with symptomatic metamorphopsia between April 2012 and March 2013 at the University Hospital and the North Medical Center at Kyoto Prefectural University of Medicine. Of the 40 patients, 2 had bilateral ERM and 38 had unilateral ERM. Symptomatic metamorphopsia was defined as a metamorphopsia score of 0.2° or higher, as detected using M-CHARTS (Inami Co., Tokyo, Japan). ERM affecting foveal morphology were confirmed by OCT in all eyes enrolled in this study. All of the enrolled patients had metamorphopsia scores of 0.2° or higher in terms of metamorphopsia of vertical line (MV) or metamorphopsia of horizontal line (MH) at baseline. Patients with a history of vitreoretinal surgery, secondary ERM, amblyopia, or other ophthalmic disorders affecting visual acuity such as glaucoma, severe cataract, keratoconus, retinal vascular occlusion and other macular disorders were excluded from the study. Patients who were unable to complete the follow-up examinations and patients with diseases involving speech-related problems, such as cerebral infarction, were excluded as well.

### Ophthalmic examinations

Ophthalmic examinations, including measurement of the best-corrected VA (BCVA), intraocular pressure, dilated-pupil fundus examination, reading ability, and metamorphopsia score, were performed for all patients at baseline and at 3, 6, and 12 months post-surgery. The baseline data were obtained for both affected and healthy eyes within 2 months before surgery. The BCVA was measured with a standard Japanese Landolt VA chart, and the decimal VA was converted to the logarithm of the minimal angle resolution (logMAR) for statistical analyses.

Reading ability was evaluated using MNREAD-J charts (Handaya Co., Tokyo, Japan), which is the Japanese-language version of the MNREAD RA charts. The MNREAD-J charts are available in two versions, each presenting different sentences consisting of 30 characters. Monocular reading ability was examined using one of the two patterns for the right eye, with the other pattern being used for the left eye. In each patient, the right eye was measured first, irrespective of whether it was affected or healthy, and reading ability was measured as previously described [7]. Briefly, the patients were asked to read the chart aloud, starting with the largest characters and continuing to read the sentences at each character size at a distance of 30 cm from the chart under appropriate near correction. The time required for reading and the frequency of errors were then recorded. The reading speed for each character size was defined as 60 × (30 – number of mistakes/time in seconds taken to read the sentence). All data were collected in a spreadsheet for calculating three parameters: 1) RA, 2) maximum reading speed (MRS), and 3) critical print size (CPS). Reading acuity was defined as the smallest character that a patient could read without significant errors, regardless of speed, while CPS was defined as the smallest character a patient can read at the MRS. The MRS was defined as the maximum number of characters that can be read in 1 min, where the character size is equal to the CPS or larger.

The severity of metamorphopsia was examined using the M-CHARTS. The Amsler test is widely used to evaluate metamorphopsia, however, it does not give the information about the severity of metamorphopsia [19]. The M-CHARTS consist of 19 dotted lines, with dot intervals ranging from 0.2° to 2.0° in terms of visual angles [20]. The metamorphopsia scores measured using the M-CHARTS included both MV and MH, as measured by presenting the chart in vertical and horizontal orientations, respectively. We evaluated by type 1 M-charts, which uses a single dotted line and is designed for patients with a fixation point such as epiretinal membrane or age-related macular degeneration [21]. As with the above-described MNREAD-J charts, the evaluation was performed with the patient sitting at a distance of 30 cm from the chart under appropriate near correction. The patients were presented with consecutive dotted lines, starting with a continuous line (0°), and then asked whether the presented line was distorted. The metamorphopsia score was defined as the smallest visual angle of the dotted line for which the patient stated that it was straight.

### Vitrectomy

All surgeries were performed using either 25G or 27G transconjunctival suture-less vitrectomy systems under local anesthesia by two experienced vitreoretinal surgeons (K.K., K.Y.). Briefly, using a wide-angle viewing system, core vitrectomy and peripheral vitreous shaving were performed. Next, after inducing posterior vitreous detachment, if not already present, the ERM and the internal limiting membrane (ILM) were removed in all cases with 0.025% indocyanine green dye staining. Simultaneous phacoemulsification and intraocular lens implantation were performed in all phakic cases to prevent any interference of cataract progression after the vitrectomy in the estimation of final vision.

### Outcome measures and statistical analysis

As outcome measures, we analyzed BCVA, reading ability with MNREAD-J, and metamorphopsia score with M-CHARTS between at baseline and at each post-surgery follow-up visit. Generally, a difference of 0.2 logMAR or more is considered a significant change in BCVA. Therefore, as a subgroup analysis, we additionally evaluated the BCVA and the reading ability of the patients who presented a BCVA difference of 0.1 logMAR or less between at baseline and at 12 month post-surgery. The Wilcoxon signed-rank test and Bonferroni correction were used for performing the statistical analyses. A *P* value of < 0.05 was considered statistically significant. All statistical analyses were performed using SAS version 9.4 statistics software (SAS Institute, Inc., Cary, NC).

## Results

Basic demographics, including patient gender, median and interquartile range (IQR) of age, and different types of visual function measurements at baseline are listed in Table 1. In all patients, the ERM was anatomically removed, and no intra- or post-surgery complications were observed.

**Table 1:** Sex and medians (IQR) of age and different types of visual-function measurements at baseline

Sex (male/female)	17/23
Patient age	69 (65–75)
BCVA (logMAR)	0.2 (0.1–0.4)
RA (logMAR)	0.3 (0.2–0.4)
CPS (logMAR)	0.8 (0.6–0.8)
MRS (characters per minute)	343 (300–381)

*IQR* interquartile range, *BCVA* best-corrected visual acuity, *logMAR* logarithm of the minimal angle resolution, *RA* reading acuity, *CPS* critical print size, *MRS* maximum reading speed

### Post-surgery changes in BCVA and reading ability

**Fig. 1:**
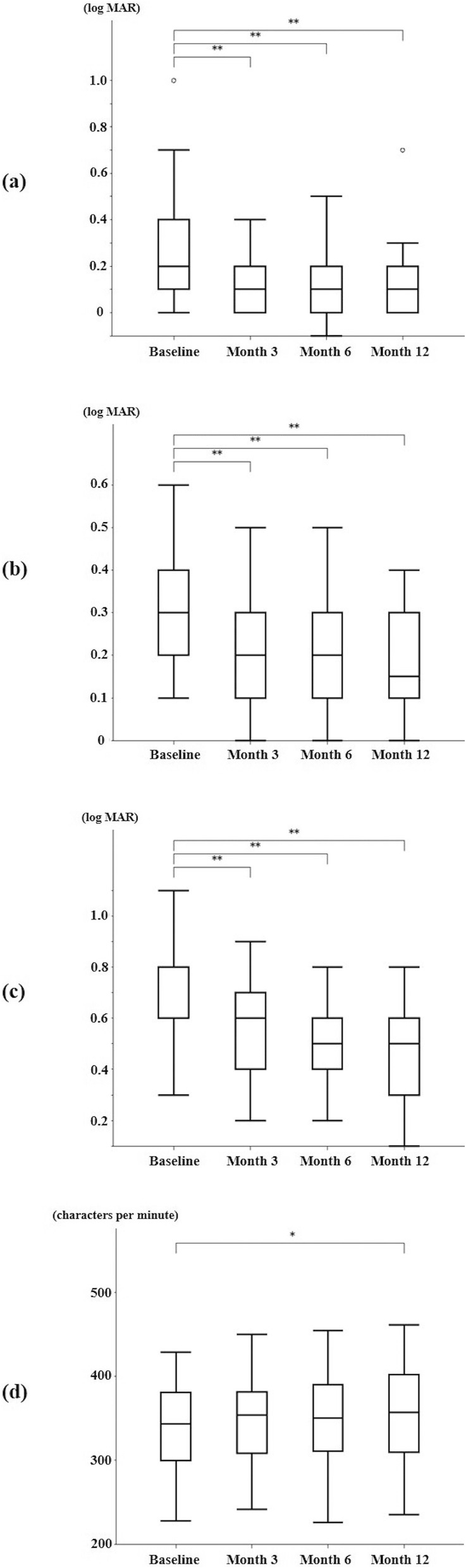
Box-whisker plots showing time-course changes in median values of **a** BCVA, **b** RA, **c** CPS, and **d** MRS during post-surgery follow-up period. Wilcoxon signed-ranks test show significant improvements in BCVA, RA, and CPS in all post-surgery follow-up examinations compared to the values at baseline (*P* < 0.001). However, the median MRS shows significant improvement only at 12 months post-surgery (*P* < 0.05). The vertical-column boxes indicate values between the 25th and 75th percentiles (central line, median). The circles denote outliers. BCVA: best-corrected visual acuity; RA: reading acuity; CPS: critical print size; MRS: maximum reading speed

**Fig. 2:**
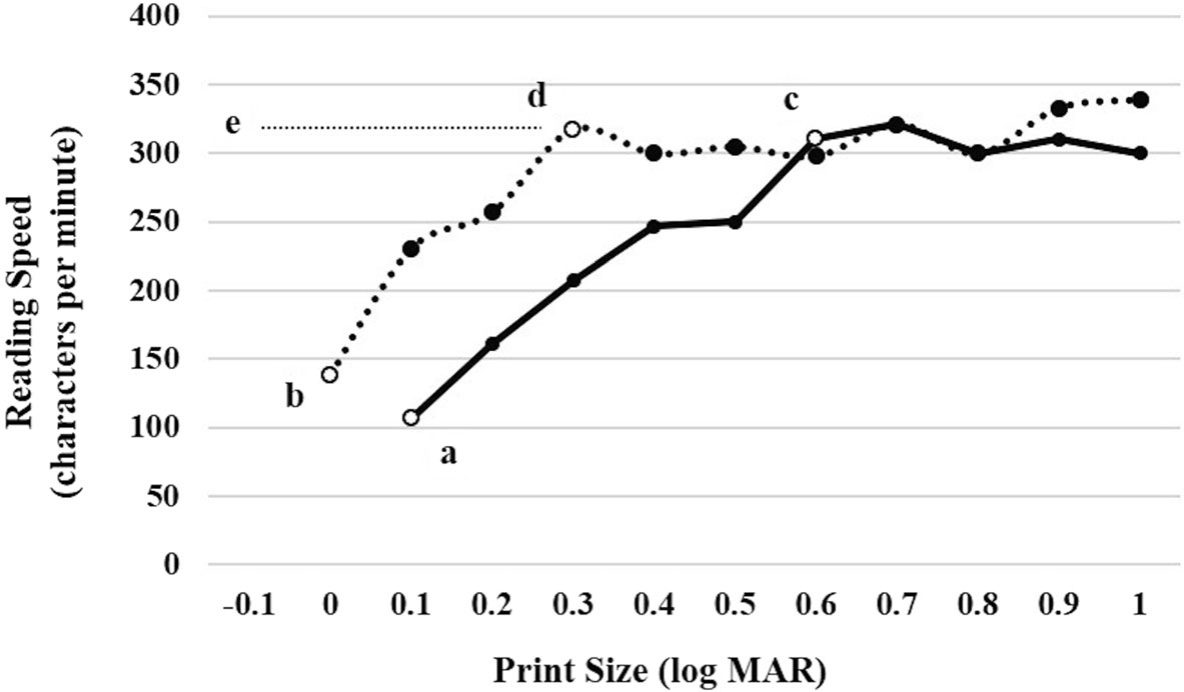
A representative case of reading ability pre- and post-surgery for ERM. The solid line indicates the reading ability before surgery, and the dashed line indicates the reading ability at 12 months post-surgery. The patient’s BCVA was 0 logMAR, both before surgery and at 12 months post-surgery. The metamorphopsia score of the vertical line improved from 1.3 at baseline to 0.5 at 12 months post-surgery, and the metamorphopsia score of the horizontal line improved from 2.0 at baseline to 0.4 at 12 months post-surgery. **a** reading acuity before surgery; **b** reading acuity at 12 months post-surgery; **c** critical print size before surgery; **d** critical print size at 12 months post-surgery; **e** maximum reading speed at 12 months post-surgery. ERM: Epiretinal membrane

The medians and IQRs of BCVA, RA, CPS, and MRS of all cases at baseline and at each post-surgery follow-up examination are summarized in Table 2. Box-whisker plots of the time courses in the median values of BCVA, RA, CPS, and MRS during the post-surgery follow-up period are shown in Fig. 1. Compared to baseline, significant improvements in median BCVA, RA, and CPS were observed at 3 months post-surgery, and these improvements were retained at each subsequent post-surgery follow-up examination (*P* < 0.001). However, significant improvement in median MRS was observed only at 12 months post-surgery (*P* = 0.04). A representative case of the change in reading ability before and after surgery is shown in Fig. 2.

**Table 2:** Median values (IQR) of different types of visual function measurements at each visit (*n* = 42)

	Baseline	3 Months Postoperative	6 Months Postoperative	12 Months Postoperative
BCVA (logMAR)	0.2 (0.1–0.4)	0.1^**^ (0–0.2)	0.1^**^ (0–0.2)	0.1^**^ (0–0.2)
RA (logMAR)	0.3 (0.2–0.4)	0.2^**^ (0.1–0.3)	0.2^**^ (0.1–0.3)	0.2^**^ (0.1–0.3)
CPS (logMAR)	0.8 (0.6–0.8)	0.6^**^ (0.4–0.7)	0.5^**^ (0.4–0.6)	0.5^**^ (0.3–0.6)
MRS (characters per minute)	343 (300–381)	354 (308–381)	350 (311–390)	357^*^ (310–402)

Statistically significant differences of each measurement compared with the values at baseline are labeled. The *P* value was calculated with the Wilcoxon signed-rank test and Bonferroni correction. ^*^P < 0.05, ^**^P < 0.001; *IQR* interquartile range, *BCVA* best-corrected visual acuity, *logMAR* logarithm of the minimal angle resolution, *RA* reading acuity, *CPS* critical print size, *MRS* maximum reading speed

**Fig. 3:**
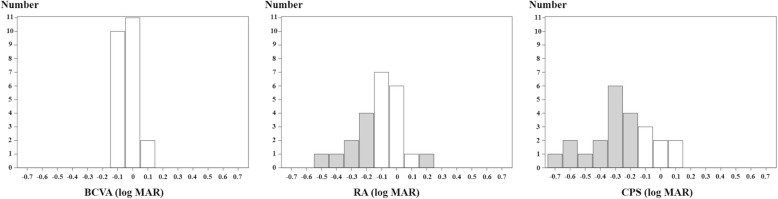
Histogram of changes in BCVA, RA, and CPS from baseline to 12 months post-surgery in 23 eyes that exhibited 0.1 logMAR or less post-surgery change in BCVA. As can be seen in the figure, the further left from 0, the greater is the degree of improvement. The white bars indicate changes smaller 0.1 logMAR, and the gray bars indicate changes greater than or equal to 0.2 logMAR. BCVA: best-corrected visual acuity; RA: reading acuity; CPS: critical print size

When compared to the values at baseline, 19 eyes showed an improvement of 0.2 logMAR or more in BCVA at 12 months post-surgery, while 23 eyes showed a change of 0.1 logMAR or less in BCVA at 12 months post-surgery. No eye exhibited a worsening of 0.2 logMAR or more in BCVA at 12 months post-surgery. Subgroup analysis of the 23 eyes that exhibited a change of 0.1 logMAR or less in BCVA at 12 months post-surgery revealed no change in the median BCVA, but both the median RA and CPS improved by 0.2 logMAR (Table 3). A histogram of the amount of change from at baseline to at 12 months post-surgery is shown in Fig. 3. As the figure shows, the degree of improvement is greater the further toward the left from 0. With regard to the BCVA of the 23 eyes, compared to the values at baseline, 11 eyes exhibited no change, 10 eyes exhibited an improvement of 0.1 logMAR, and 2 eyes exhibited a deterioration of 0.1 logMAR at 12 months post-surgery. Compared to the BCVA, RA and CPS further improved in many patients after surgery.

**Table 3:** Median values (IQR) of RA and CPS at baseline and 12 months post-surgery of 23 eyes with a BCVA change of 0.1 logMAR or less

	Baseline	12 Months Postoperative
BCVA (logMAR)	0.1 (0.1–0.2)	0.1 (0–0.1)
RA (logMAR)	0.3 (0.2–0.4)	0.1 (0.1–0.2)
CPS (logMAR)	0.7 (0.6–0.8)	0.5 (0.4–0.5)

*IQR* interquartile range, *RA* reading acuity, *CPS* critical print size, *logMAR* logarithm of the minimal angle resolution, *BCVA* best-corrected visual acuity

### Changes in metamorphopsia score

The median and IQR of the metamorphopsia score are listed in Table 4. No significant change in the MV score was observed throughout the post-surgery follow-up period. However, and compared to the value at baseline, a significant improvement in the MH score was observed at 3, 6 (*P* < 0.05), and 12 months (*P* < 0.001) post-surgery.

**Table 4:** Median values (IQR) of metamorphopsia score in each visit (*n* = 42)

	Baseline	3 Months Postoperative	6 Months Postoperative	12 Months Postoperative
MV	0.7 (0.4–1.6)	0.7 (0.4–1.6)	0.7 (0.3–1.2)	0.7 (0.5–0.9)
MH	0.8 (0.4–1.5)	0.6^†^ (0–1.1)	0.4^†^ (0–1.0)	0.5^††^ (0–0.8)

Statistically significant differences in each measurement compared with the baseline were labeled. *P* values were calculated with the Wilcoxon signed-rank test and Bonferroni correction. ^†^*P* < 0.05, ^††^*P* < 0.001; *IQR* interquartile range, *MV* metamorphopsia of vertical line, *MH* metamorphopsia of horizontal line

## Discussion

In this study, our findings revealed that after surgical removal of the ERM, BCVA, reading ability, and metamorphopsia scores improved significantly compared to the baseline. It was reported that surgery for a macular hole and ERM improved retinal sensitivity and reading ability as evaluated with MNREAD [6]. We obtained similar results in the present study, but further analysis of the subgroups provided a new insight that ERM surgery might improve reading ability to a greater extent than it improves VA. In addition, compared to the findings reported in [6], the baseline BCVA and the reading ability of the patients in this study were better. Our findings of improved reading ability, together with the previously reported improvement in contrast sensitivity [22], parafoveal retinal sensitivity [23], and stereopsis [24], suggest that along with the increased widespread use of OCT and MIVS in the clinical setting, it is justifiable to expand the surgical indication of ERM when compared with the previous standard of 20G vitreous surgery.

Among the reading ability parameters measured with MNREAD, the most critical factor influencing the daily lives of patients is the CPS because it refers to the smallest font size that a patient can read at his or her optimal reading speed, that is, the MRS. Reportedly, a smaller CPS value means better reading performance, and it indicates that the patient requires a lower level of magnification to read comfortably [25]. It has been reported that MRS is specific to each individual, usually in accordance with their age [13, 26, 27], and that RA has a weaker effect on daily life because RA is the smallest font size that the patient is able to read. In the present study, although the MRS increased significantly at 12 months post-surgery, its median value changed by approximately 4% from 343 characters per minute (CPM) to 357 CPM, which is considered to be of poor clinical significance. By contrast, the CPS improved by 0.2 logMAR, not only in all of the enrolled subjects but also in the cases with a BCVA change of 0.1 logMAR or less at 12 months post-surgery, which is considered to be of notable clinical significance. This finding suggests that ERM removal improves reading ability in daily life, even if it does not improve BCVA. In addition, it indicates that performing visual-function tests other than BCVA is necessary for assessing the surgical results of ERM removal, which is currently being performed in patients with better BCVA.

Based on assessments using M-CHARTS, studies have reported that metamorphopsia improved post ERM removal [2, 28, 29]. In the examinations conducted herein, no significant improvement in the MV score was observed, but the MH score improved significantly during the post-surgery follow-up period. In a previous study, it was reported that humans were better at detecting horizontal lines than they were at detecting vertical lines and that the MH score deteriorated significantly more than the MV score as the stage of the disease advanced [20]. Moreover, it was reported that the MV score was less likely to improve compared to the MH score [29]. In the present study, the median MH score deteriorated to a greater extent than the median MV score at baseline, and the MH score improved to a greater extent than the MV score post-surgery, as has been reported previously.

Notably, the present study does have a few limitations. The first limitation is the relatively small sample size, especially in the sub-group analysis of patients. This was because we had to choose eyes with relatively small differences in VA. Second, because we did not find a correlation between the metamorphopsia score and the reading ability parameters, we were unable to specify the factor explaining the deterioration in reading ability in ERM. We did not find a correlation between the metamorphopsia score and BCVA either. Given that ERM surgery reportedly improves not only metamorphopsia but also contrast sensitivity [22], parafoveal retinal sensitivity [23], and stereopsis [24], we were unable to elucidate whether the improvement in CPS is caused by any one of those factors or by multiple factors. Third, in this study, we did not include a correlation between reading ability parameters and structural retinal changes. Thus, studies should be conducted to clarify why CPS was the most significant factor related to the visual changes observed from pre to post vitreous surgery for ERM.

## Conclusions

Our findings indicated that the surgical removal of ERM improved reading ability, even when the BCVA was not improved. The measurement of reading performance appears to be a helpful additional test to VA as a measure for the assessment of ERM surgery.

## Data Availability

The datasets used in the current study are available from the corresponding author upon reasonable request.

## Abbreviations

BCVA: Best-corrected visual acuity
CPM: Characters per minute
CPS: Critical print size
ERM: Epiretinal membrane
G: Gauge
ILM: Internal limiting membrane
IQR: Interquartile range
logMAR: Minimal angle resolution
MH: Metamorphopsia of horizontal line
MIVS: Micro-incision vitreous surgery
MNREAD: Minnesota Reading
MRS: Maximum reading speed
MV: Metamorphopsia of vertical line
OCT: Optical coherence tomography
PPV: Pars plana vitrectomy
RA: Reading acuity
VA: Visual acuity
